# Supplementary material for: Detection and phylogenetic characterization of Jingmen tick virus in Amblyomma mixtum ticks from Costa Rica
Source: Microbiol Spectr. 2026 May 13;14(6):e04078-25. doi: 10.1128/spectrum.04078-25 (PMC13227955; doi:10.1128/spectrum.04078-25)
Supplement: Supplemental material — Fig. S1; Tables S1 to S5. [file spectrum.04078-25-s0001.pdf]

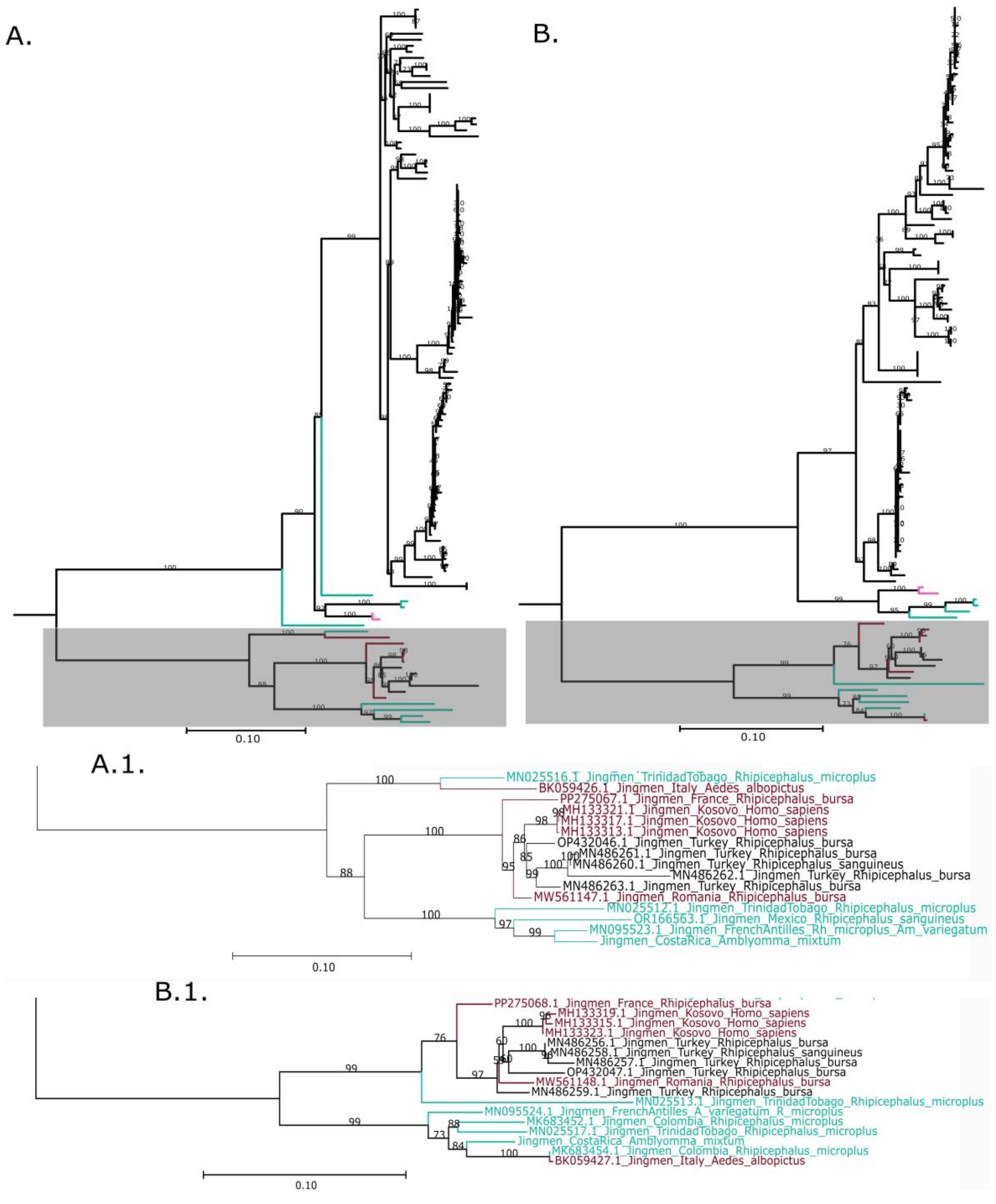

Supplementary Figure. Phylogenetic trees of: **A.** segment 1 encoding for the RdRp, including JMTV Mexico, and B. segment 2 encoding for the glycoprotein, including JMTV Colombia. Cropped regions from the subclade CII are shown in A.1 (segment

1) and **B.1.** (segment 2). MAGs from JMTV Costa Rica were assembled in CLC genomics from three tick pools collected from the same collection site. Other 118 sequences for each segment were downloaded from the Genbank Database. Alignments were performed in MAFFT and trimmed in MEGA 12. Trees were calculated with IQTree and ModelFinder (best fit model for each segment: 1: TIM3+F+R3, and segment 2: TNe+R3). Bootstraps were estimated with 1000 replicates, and approximate Bayes Test, and are shown in each branch. Midpoint rooted trees were visualized in TreeViewer and Inkscape. Sequences are color-coded by region of origin.

Supplementary Table 1. Nucleotide pairwise distance matrices comparing the consensus sequences obtained for each genome segment in each tick pool.

		am04	am05	am06
Segment 1 Consensus length 2472 nt	am04			
	am05	0,000815		
	am06	0,000809	0,000407	
Segment 2 Consensus length 2025 nt	am04			
	am05	0,002		
	am06	0,001	0,003	
Segment 3 Consensus length 1881 nt	am04			
	am05	0,001		
	am06	0,000	0,001	
Segment 4 Consensus length 426 nt	am04			
	am05	0,002		
	am06	0,000	0,002	

Supplementary Table 2. Nucleotide pairwise distance matrix for the segment 1 encoding the RNA dependent RNA polymerase for Jingmen tick viruses of the subclade CII.

	CostaRica	MN095523.1	MN025512.1	MN025516.1	BK059426.1	PP275067.1	MH133321.1	MH133317.1	MH133313.1	MW561147.1	OP432046.1	MN486261.1	MN486260.1	MN486263.1
CostaRica_Amblyomma_mixtum														
MN095523.1_FrenchAntilles_R_microplus_A_variegatum	0.037176													
MN025512.1_TrinidadTobago_Rhipicephalus_microplus	0.100663	0.091220												
MN025516.1_TrinidadTobago_Rhipicephalus_microplus	0.155345	0.151152	0.169082											
BK059426.1_Italy_Aedes_albopictus	0.157968	0.158770	0.168414	0.070497										
PP275067.1_France_Rhipicephalus_bursa	0.172545	0.159671	0.175231	0.161127	0.171349									
MH133321.1_Kosovo_Homo_sapiens	0.166086	0.159211	0.174891	0.158828	0.163908	0.053923								
MH133317.1_Kosovo_Homo_sapiens	0.165539	0.158673	0.174336	0.159855	0.164456	0.053910	0.000729							
MH133313.1_Kosovo_Homo_sapiens	0.165539	0.158673	0.174336	0.160308	0.163994	0.054300	0.002555	0.001823						
MW561147.1_Romania_Rhipicephalus_bursa	0.170102	0.160457	0.175849	0.162570	0.171353	0.060204	0.041740	0.041730	0.041346					
OP432046.1_Turkey_Rhipicephalus_bursa	0.166180	0.156873	0.173854	0.161846	0.173966	0.064728	0.037519	0.037510	0.037128	0.031419				
MN486261.1_Turkey_Rhipicephalus_bursa	0.170169	0.164696	0.176484	0.166967	0.174676	0.063249	0.041433	0.041030	0.041415	0.036828	0.033017			
MN486260.1_Turkey_Rhipicephalus_sanguineus	0.170292	0.163817	0.175598	0.164922	0.173700	0.063997	0.041422	0.041413	0.041797	0.036038	0.031471	0.002923		
MN486263.1_Turkey_Rhipicephalus_bursa	0.172047	0.163266	0.176597	0.161807	0.171636	0.063006	0.039171	0.039965	0.038811	0.035339	0.032685	0.034245	0.033443	
MN486262.1_Turkey_Rhipicephalus_bursa	0.202380	0.197571	0.207073	0.200865	0.211491	0.099884	0.076651	0.076632	0.077039	0.075471	0.072283	0.045095	0.042704	0.069812

Supplementary Table 3. Nucleotide pairwise distance matrix for the segment 2 encoding the glycoprotein for Jingmen tick viruses of the subclade CII.

	CostaRica	MN095524.1	MN025517.1	MN025513.1	BK059427.1	PP275068.1	MH133319.1	MH133323.1	MH133315.1	MN486256.1	MN486258.1	MW561148.1	OP432047.1	MN486259.1
CostaRica_Amblyomma_mixtum														
MN095524.1_ FrenchAntilles_A_variegatum_R_microplus	0.0725													
MN025517.1_ TrinidadTobago_Rhipicephalus_microplus	0.0675	0.0720												
MN025513.1_ TrinidadTobago_Rhipicephalus_microplus	0.1793	0.1708	0.1868											
BK059427.1_ Italy_Aedes_albopictus	0.0844	0.0786	0.0896	0.1741										
PP275068.1_ France_Rhipicephalus_bursa	0.1571	0.1457	0.1607	0.1084	0.1486									
MH133319.1_ Kosovo_Homo_sapiens	0.1592	0.1509	0.1619	0.1105	0.1479	0.0523								
MH133323.1_ Kosovo_Homo_sapiens	0.1569	0.1487	0.1595	0.1110	0.1479	0.0508	0.0031							
MH133315.1_ Kosovo_Homo_sapiens	0.1593	0.1499	0.1608	0.1088	0.1491	0.0537	0.0031	0.0027						
MN486256.1_ Turkey_Rhipicephalus_bursa	0.1652	0.1563	0.1661	0.1186	0.1550	0.0562	0.0392	0.0368	0.0397					
MN486258.1_ Turkey_Rhipicephalus_sanguineus	0.1647	0.1569	0.1656	0.1180	0.1545	0.0567	0.0397	0.0373	0.0401	0.0013				
MW561148.1_ Romania_Rhipicephalus_bursa	0.1689	0.1609	0.1739	0.1168	0.1587	0.0527	0.0393	0.0378	0.0397	0.0435	0.0450			
OP432047.1_ Turkey_Rhipicephalus_bursa	0.1735	0.1651	0.1732	0.1152	0.1624	0.0559	0.0370	0.0355	0.0384	0.0422	0.0436	0.0394		
MN486259.1_ Turkey_Rhipicephalus_bursa	0.1628	0.1522	0.1642	0.1136	0.1524	0.0563	0.0375	0.0365	0.0370	0.0399	0.0395	0.0471	0.0492	
MN486257.1_ Turkey_Rhipicephalus_bursa	0.2041	0.1914	0.2040	0.1584	0.1903	0.0956	0.0783	0.0757	0.0777	0.0409	0.0394	0.0840	0.0816	0.0688

Supplementary Table 4. Nucleotide pairwise distance matrix for the segment 3 encoding the NS3-like protein for Jingmen tick viruses of the subclade CII.

	BK059428 .1	MN095525 .1	Costa Rica	MN025518 .1	MN025514 .1	MH133314 .1	MH133318 .1	MH133322 .1	MN486265 .1	MN486264 .1	MW561149 .1	OP432048 .1	MN486266 .1	PP275069 .1
BK059428.1_Italy_Aedes_ albopictus														
MN095525.1_FrenchAntilles_ R_microplus_A_variegatum	0.03042													
CostaRica_Amblyomma _mixtum	0.04315	0.01881												
MN025518.1_TrinidadTobago_ Rhipicephalus_microplus	0.06616	0.05692	0.06617											
MN025514.1_TrinidadTobago_ Rhipicephalus_microplus	0.13433	0.12509	0.13161	0.12631										
MH133314.1_Kosovo_Homo_ sapiens	0.14870	0.14062	0.14164	0.14357	0.15659									
MH133318.1_Kosovo_Homo_ sapiens	0.14924	0.14115	0.14217	0.14561	0.15718	0.00621								
MH133322.1_Kosovo_Homo_ sapiens	0.14927	0.14118	0.14220	0.14565	0.15722	0.00704	0.00248							
MN486265.1_Turkey_ Rhipicephalus_sanguineus	0.15104	0.14134	0.14453	0.14538	0.16285	0.02780	0.02736	0.02737						
MN486264.1_Turkey_ Rhipicephalus_bursa	0.15421	0.14346	0.14666	0.14752	0.16397	0.02780	0.02822	0.02823	0.00248					
MW561149.1_Romania_ Rhipicephalus_bursa	0.14782	0.13611	0.13825	0.13895	0.15586	0.02823	0.02736	0.02737	0.02827	0.02913				
OP432048.1_Turkey_ Rhipicephalus_bursa	0.14994	0.13620	0.13776	0.14221	0.15632	0.03862	0.03818	0.03732	0.03784	0.03871	0.03131			
MN486266.1_Turkey_ Rhipicephalus_bursa	0.16228	0.15128	0.15295	0.15429	0.16721	0.04687	0.04644	0.04733	0.03720	0.03897	0.04294	0.05144		
PP275069.1_France_ Rhipicephalus_bursa	0.14713	0.14058	0.14425	0.14190	0.15104	0.05415	0.05281	0.05371	0.05510	0.05600	0.04932	0.05603	0.06324	
MN486271.1_Turkey_ Rhipicephalus_bursa	0.19807	0.18917	0.19147	0.19192	0.21131	0.07258	0.07069	0.07070	0.04460	0.04730	0.07169	0.08260	0.07349	0.10233

Supplementary Table 5. Nucleotide pairwise distance matrix for the segment 4 encoding the membrane protein for Jingmen tick viruses of the subclade CII.

	Costa Rica	BK059429.1	MN095526.1	MN025519.1	MN025515.1	MH133316.1	MH133320.1	MH133324.1	MN486269.1	MN486267.1	MN486270.1	MN486268.1	MW561150.1	OP432049.1
CostaRica_Amblyomma_mixtum														
BK059429.1_Italy_Aedes_albopictus	0.07057													
MN095526.1_FrenchAntilles_R_microplus_A_variegatum	0.07298	0.04549												
MN025519.1_TrinidadTobado_Rhipicephalus_microplus	0.05933	0.07038	0.06908											
MN025515.1_TrinidadTobago_Rhipicephalus_microplus	0.12716	0.13093	0.12637	0.12286										
MH133316.1_Kosovo_Homo_sapiens	0.15008	0.16049	0.15382	0.15416	0.15337									
MH133320.1_Kosovo_Homo_sapiens	0.14919	0.15959	0.15292	0.15161	0.15082	0.00199								
MH133324.1_Kosovo_Homo_sapiens	0.14767	0.15806	0.15140	0.15174	0.15096	0.00534	0.00333							
MN486269.1_Turkey_Rhipicephalus_sanguineus	0.13761	0.14881	0.14533	0.14581	0.14738	0.04117	0.03905	0.03697						
MN486267.1_Turkey_Rhipicephalus_bursa	0.14007	0.15129	0.14780	0.14663	0.14655	0.04188	0.03975	0.03767	0.00199					
MN486270.1_Turkey_Rhipicephalus_bursa	0.14563	0.14945	0.14256	0.14481	0.15068	0.04119	0.03907	0.03838	0.01683	0.01751				
MN486268.1_Turkey_Rhipicephalus_bursa	0.15025	0.16003	0.15647	0.15610	0.15586	0.05657	0.05439	0.05226	0.01761	0.01693	0.03011			
MW561150.1_Romania_Rhipicephalus_bursa	0.14929	0.15636	0.15470	0.15600	0.15087	0.04469	0.04256	0.04188	0.03267	0.03337	0.03341	0.04639		
OP432049.1_Turkey_Rhipicephalus_bursa	0.14444	0.14653	0.14636	0.14766	0.15634	0.05034	0.04819	0.04539	0.03824	0.04034	0.04461	0.05349	0.04038	
PP275070.1_France_Rhipicephalus_bursa	0.14109	0.15562	0.14796	0.15015	0.14968	0.06137	0.05918	0.05705	0.05195	0.05267	0.05631	0.06770	0.05487	0.06342
